# Pulmonary ventilation–perfusion mismatch: a novel hypothesis for how diving vertebrates may avoid the bends

**DOI:** 10.1098/rspb.2018.0482

**Published:** 2018-04-25

**Authors:** Daniel Garcia Párraga, Michael Moore, Andreas Fahlman

**Affiliations:** 1 Fundación Oceanogràfic, Ciudad de las Artes y las Ciencias, 46013 Valencia, Spain; 2 Woods Hole Oceanographic Institution, Woods Hole, MA 02543, USA

**Keywords:** diving physiology, cardiorespiratory physiology, whale stranding, noise pollution, climate change

## Abstract

Hydrostatic lung compression in diving marine mammals, with collapsing alveoli blocking gas exchange at depth, has been the main theoretical basis for limiting N_2_ uptake and avoiding gas emboli (GE) as they ascend. However, studies of beached and bycaught cetaceans and sea turtles imply that air-breathing marine vertebrates may, under unusual circumstances, develop GE that result in decompression sickness (DCS) symptoms. Theoretical modelling of tissue and blood gas dynamics of breath-hold divers suggests that changes in perfusion and blood flow distribution may also play a significant role. The results from the modelling work suggest that our current understanding of diving physiology in many species is poor, as the models predict blood and tissue N_2_ levels that would result in severe DCS symptoms (chokes, paralysis and death) in a large fraction of natural dive profiles. In this review, we combine published results from marine mammals and turtles to propose alternative mechanisms for how marine vertebrates control gas exchange in the lung, through management of the pulmonary distribution of alveolar ventilation (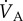) and cardiac output/lung perfusion (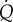), varying the level of 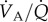 in different regions of the lung. Man-made disturbances, causing stress, could alter the 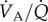 mismatch level in the lung, resulting in an abnormally elevated uptake of N_2_, increasing the risk for GE. Our hypothesis provides avenues for new areas of research, offers an explanation for how sonar exposure may alter physiology causing GE and provides a new mechanism for how air-breathing marine vertebrates usually avoid the diving-related problems observed in human divers.

## 1. General overview on respiratory anatomy and physiology in marine mammals

While exposure to high pressure is a common challenge among breath-hold divers, there is large variation in respiratory anatomy, function and capacity between genera and even species [1–3]. The ultra-deep-diving feats of some marine mammals go beyond our current understanding of respiratory physiology and lung mechanics. Many diving adaptations relate to behaviour, intermittent ventilation and physiological functions that support simultaneous activities, including gas exchange, aerobic metabolism, buoyancy control and air storage for echolocation, social vocalization and foraging [1,2,4]. As a generalization, deep divers tend to have greater overall O_2_ stores, with a greater proportion of muscle mass, higher myoglobin content, a greater blood volume and higher haematocrit [5–8]. Lungs of deep-diving whales have a smaller mass-specific total lung capacity, are typically smaller, occupy a smaller percentage of thoracic volume and are associated with more vascular retia than the lungs of shallower divers such as nearshore bottlenose dolphins [9,10]. Increased lung volume while diving could increase the amount of N_2_ taken up during dives, and thereby increase the risk of decompression sickness (DCS) [11,12]. Thus in deep-diving species, available O_2_ is mainly augmented by increased haemoglobin and blood volume as well as muscle myoglobin [8,9,13].

In cetaceans, the cartilaginous reinforcements that maintain patency of the airways during diving serve different goals, including facilitation of high respiratory flow and short breath durations at the surface. Furthermore, the airway reinforcement associated with a greater lung compliance [1] facilitates alveolar collapse, limiting N_2_ absorption during dives. The reinforcements also help to maintain airway permeability (no gas trapping) during compression, and provide an air storage site in a non-gas-exchange compartment when lung parenchyma collapses at depth [1,2]. At least in some pinnipeds the airways compress to some extent during a dive [14], but no study has measured airway compression in a live cetacean. In 1969, Ridgway *et al*. [15] showed in bottlenose dolphins that O_2_ content in the first breath after a 300 m dive was greater than after an equivalent amount of horizontal swimming at 20 m. This finding supports the theory that alveolar collapse and air displacement into the reinforced trachea and primary bronchi [10], or terminal bronchi [5,14,16], help reduce exchange of gases across the alveolar–capillary barrier during diving. McDonald & Ponganis [17] provided direct evidence of alveolar collapse during descent and alveolar recruitment during ascent in freely diving sea lions. However, the extent of compression of the tracheobronchial tree on different species is still debated [10,11,18].

Besides airway reinforcement, recent studies on cetaceans have demonstrated the presence of vascular plexuses (mainly venous) along the airways (well extended into the terminal bronchus in deep-diving whales) [4,16,19–22]. It has been hypothesized that engorgement of the plexus during dives would help to reinforce the airways during dives [3,4]. They could also help warm the air, serve as an oxygen store, reduce the internal volume of the airway and prevent extreme intraluminal negative pressures minimizing deformity of the tracheal wall [19–21]. This mechanism could also assure that reduced air volume at great depths is displaced into the sound-producing areas, structures not typically covered with venous plexuses.

## 2. Present perspective: passive alveolar collapse as the main driving force to minimize nitrogen uptake

In an early study, Scholander [10] argued that passive compression of the highly compliant alveolus and rigid conducting airways would result in cessation of gas exchange, which would prevent N_2_ uptake and reduce the risk of gas emboli (GE). Until recently, this lung compression/alveolar collapse model has been the main hypothesis as to how marine mammals prevent excessive uptake of N_2_ and avoid diving-related complications, such as DCS and N_2_ narcosis. However, it is not known how they prevent excessive N_2_ uptake during dives shallower than the estimated alveolar collapse depth. In addition, it was proposed that changes in cardiac output (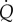) would alter N_2_ exchange [23], and theoretical modelling studies agree that variation on perfusion and blood flow distribution will have a marked effect on blood and tissue N_2_ levels [11,24–26]. Here, we aim to summarize past studies that have attempted to assess how lung compression and atelectasis alter gas dynamics. From this synthesis, we provide an alternative perspective as to how cetaceans, and possibly other marine mammals, and sea turtles, may manage a pulmonary shunt through alteration in alveolar ventilation (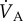) and perfusion (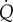) that allows selective gas exchange during natural dives. This may help explain how stressful disturbances could cause failure of these evolved mechanisms, leading to GE and even DCS.

Research to establish the alveolar collapse depth in cetaceans has been used to predict how these animals avoid DCS, and has been studied in various ways. In the bottlenose dolphin, muscle N_2_ washout following more than 20 repeated dives to 100 m was used to estimate the alveolar collapse depth at 70 m [27]. A recent analysis of these data provide an alternative conclusion suggesting that the actual alveolar collapse depth is highly variable and is probably considerably deeper [24]. Another study imaged marine mammal cadavers in a fibreglass pressure chamber at different pressures. The lung volumes at different pressures were estimated from three-dimensional reconstruction of the images, allowing the depth of alveolar collapse to be estimated [28]. While it was admitted that post-mortem changes could bias the results, the estimated alveolar collapse depth was deeper when compared with estimates from other investigations [27,29]. Houser *et al.* [30] measured venous blood N_2_ levels in bottlenose dolphins during the post-dive period after a series of 10–12 dives to 100 m and did not find any elevation of the blood N_2_ tension (PN_2_). These results do not necessarily contradict the findings by Ridgway & Howard [27] as a different compartment was sampled and the dive series was shorter in the more recent study.

The actual degree of pulmonary shunt has been determined for some pinniped species [12], but not in cetaceans. In the harbour seal and California sea lion, the pulmonary shunt at the surface was 8% and 13%, respectively. The shunt progressively increased to 70% at 10 ATA in the seal, and was 57% in the sea lion at 7.8 ATA. Based on these data, complete alveolar collapse was estimated at 170 m for the harbour seal and at 160 m for the sea lion [12]. Further evidence for alveolar collapse and pulmonary shunt has been demonstrated through measurement of arterial O_2_ tension (PO_2_) in free-diving California sea lions [17]. During deep dives (300–400 m) the arterial PO_2_ abruptly declined at approximately 200 m during descent, and increased at the same depth on ascent. A rise in arterial PO_2_ during ascent associated with an increase in heart rate, most probably reflecting an increase in 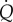, contributed to a second rise in arterial PO_2_ during the last portion of the dive.

**Figure 1.**
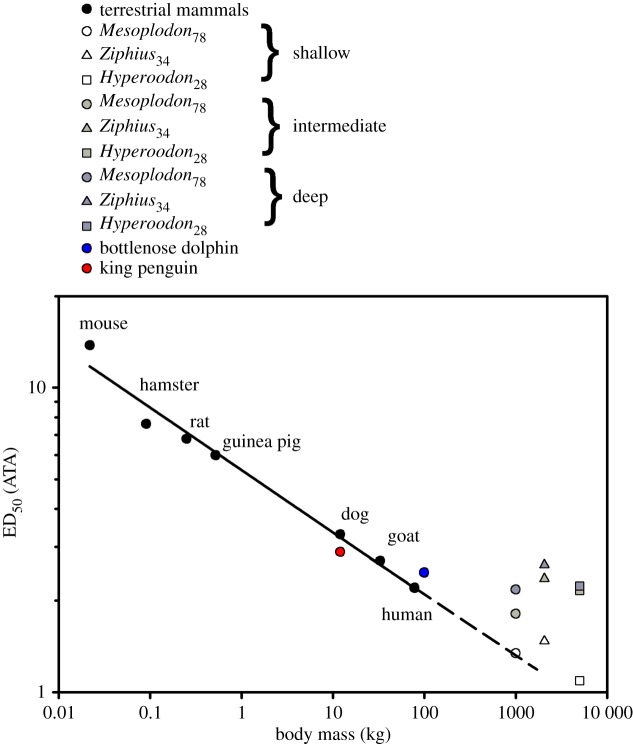
The median N_2_ saturation/mixed venous inert gas tension (PvN_2_) pressure (ED_50_) causing in 50% severe decompression sickness (DCS) in a range of terrestrial mammals following rapid decompression [37,38]. Also included are the estimated end-dive PvN_2_ for a range of breath-hold divers using present gas dynamics models [11,24–26,39]. Black circles are PvN_2_ for terrestrial animals. The solid line indicates the best-fit regression logED_50_ = 0.730–0.205 log*M*_b_. Figure reproduced from Hooker *et al*. [26]. Open and grey symbols are average PvN_2_ for Blainville's beaked whale, Cuvier's beaked whale and northern bottlenose whale at shallow (greater than 5 m ≤ 50 m), intermediate (greater than 50 m ≤ 200 m) and deep (greater than 200 m) depths [26]. Blue circle is the estimated PvN_2_ for the king penguin [25], and red is that for the bottlenose dolphin (Fahlman *et al*. 2018, unpublished observation). Note how estimated PvN_2_ at the end of each dive are high enough to lead these animals to suffer DCS in 50% of their dives or even more, which implies that some other mechanisms should exist to help minimize N_2_ uptake.

The alveolar collapse depth is affected by the structural properties of the respiratory system, and the ratio between alveolar and airway dead space volume and therefore the diving lung volume [10,11,18]. Deep divers are reported to have comparatively smaller lung volumes when compared with shallow-diving species, which is thought to reduce N_2_ uptake and the risk for GE [2,5]. Voluntary adjustment of the initial diving lung volume could adjust O_2_ stores or minimize N_2_ uptake, and hence the risk of GE [11,17,31]. Individual variation in such adjustments would mean that alveolar collapse depth and thus the potential risk of DCS in case of altered behaviour would change [1]. The degree of pulmonary shunt, through lung compression, probably increases with depth as demonstrated experimentally [12] and predicted theoretically [11,18,32]. Modelling studies have shown how theoretical estimations may provide useful insights into complex physiological systems, where a number of studies with seemingly varying results can be explained on the basis of a unifying theory [10,15,17,27,29,33–35]. However, it is important to realize that results from these theoretical constructs, such as estimating blood and tissue gas distribution during diving, are limited by available information about basic cardiorespiratory physiology (e.g. pulmonary and cardiac shunt fractions, gas diffusion coefficient through alveolar membranes under compression and total inspired air volume before submersion), the structural properties of the various portions of the respiratory system (e.g. respiratory compliance) [35], and the link between ventilation (alveolar ventilation, 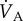) and 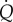 in live animals [11,18]. Considering all present knowledge and assuming passive compression of the respiratory system to achieve complete alveolar collapse, complex theoretical lung compression models are still unable to explain how marine mammals avoid the bends [26,36]. The estimated N_2_ tensions at the end of routine dives in a number of marine vertebrates from the current models suggest that the probability of experiencing severe DCS is 50% or more (figure 1). Thus, these models highlight that (1) the physiological responses during diving are not very well understood and (2) there are likely mechanisms that help reduce these unlikely levels of blood and tissue N_2_ levels. Based on past studies, and recent work in the loggerhead sea turtle (*Caretta caretta*) [40], we propose that a combination of a variety of cardiorespiratory traits permit active control on 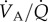 matching, allowing selective gas exchange at almost any depth. We will first discuss evidence of GE in marine mammals and then the case of the sea turtle. Finally, we will provide additional data to make a general case as to how volitional 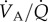 regulation could be a common trait to manage gas exchange in cetaceans in particular, but also as a general mechanism in other breath-hold divers.

## 3. Evidence for gas embolism and decompression sickness in breath-hold divers

Cases of excessive GE associated with lesions have been reported in stranded cetaceans, particularly in beaked whales adjacent to naval exercises where mid-frequency sonar was used [41,42]. Pathologic findings consistent with dysbaric osteonecrosis in sperm whales also raised the question of potential GE [43,44]. How marine mammals manage gas exchange during diving is critical to understand how they avoid diving-related problems such as hypoxia, N_2_ narcosis and DCS, and this information is vital to understand how anthropogenic disturbances and environmental change will affect breath-hold diving vertebrates and provide important guidance for conservation efforts. Sonar-related mass strandings have increased research efforts looking for evidence of gas bubbles, as well as theoretical modelling of N_2_ uptake and distribution in an attempt to understand potential risk factors [45]. Recently, a study found two Risso's dolphins with systemic GE most probably caused by intense stress at depth associated with large prey capture causing alteration in diving behaviour and physiology [46]. Interestingly, a similar case of asphyxiation was reported in a dolphin [47], but the paper did not mention whether the necropsy included checking for gas bubbles. However, the author confirmed that no GE was detected (N. Stephens 2018, personal communication). Decompression gas bubbles were also detected in 65% of carcasses of seals and dolphins drowned at depth in gill nets [48,49], and also observed in 55% of bycaught sea turtles entrapped in gill nets or trawlers [50,51]. In marine mammals, these results have been considered evidence of blood N_2_ supersaturation during dives [48], similar to results observed by Kooyman in his pressure chamber 37 years earlier [2,33]. However, at least in turtles, the situation of entrapment in fishing gear does not necessarily reflect what occurs during a routine dive [51]. Bycaught sea turtles can show signs of DCS after entrapment even at shallower depths, during shorter immersion times and slower ascent rates than the normal limits that could take place during natural dives [50]. Trying to elucidate the potential mechanism, we investigated the functional characteristics of the pulmonary arterial vessels (particularly pulmonary sphincters at the pulmonary arteries), and lung parenchyma that could clarify the pathophysiology of GE in the sea turtle. Our results, and those from our ongoing work on marine mammal lung function, have resulted in a new hypothesis that we believe may explain how marine mammals manage gases during diving, and how failure of this mechanism due to man-made or even some natural disturbances could alter gas exchange and increase the risk of DCS.

## 4. Sea turtles as an animal model to show how ventilation and perfusion matching can be used to minimize nitrogen uptake in breath-hold divers

In a recent study, it was reported that bycaught loggerhead sea turtles experienced varying degrees of GE after being hauled to the surface [50,51]. In the 2014 study, where DCS was diagnosed by reversal of clinical signs by recompression, it was proposed that the muscular section of the pulmonary artery, the pulmonary sphincter, could play a role in the mechanism of how turtles manage gases. We therefore investigated the vasoactive properties of the arterial vessels (pulmonary and systemic), and the lung parenchyma in loggerhead sea turtles (*Caretta caretta*) and also in the red-eared slider fresh water turtle (*Trachemys scripta elegans*) [40]. During diving there is elevated parasympathetic tone that helps reduce the heart rate. When the arterial vessels and lung parenchyma were exposed to carbachol, a parasympathetic agonist, the tissues contracted in both species (see electronic supplementary material, S1). When the vessels and tissues were exposed to epinephrine (sympathetic response), all vessels contracted in the red-eared slider turtle, but pulmonary arteries and especially at the region of the pulmonary sphincters relaxed in the sea turtle. From these results, we propose that the parasympathetic tone that prevails during diving strongly constricts the pulmonary arterial sphincter, causing a pulmonary shunt that limits pulmonary perfusion and gas exchange. The sphincter relaxes by sympathetic stimulation, which could happen when submerged sea turtles become entrapped in fishing gear at depth and start fighting against the gear [50]. This finding provides a mechanism for how disruption of the normal dive response may increase N_2_ uptake through increased lung perfusion, resulting in the formation of blood and tissue GE in turtles. Consequently, we hypothesize that the development of GE in this species is caused by failure of the protective physiological mechanism that creates a shunt as the turtle tries to escape from the fishing gear. We propose that a similar mechanism may also exist in cetaceans where stress may cause failure to manage the 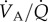 mismatch that controls exchange of gases. In contrast with the studies of sonar-exposed cetaceans, bycaught marine mammals [48] or cases of live stranded dolphins [52], bubbles in healthy *Tursiops* were not detectable on ultrasound in the portal vein of animals trained to do serial dives at 100 m depth during Houser's studies [30], nor in any of the dolphins examined after capture for health exams [53], nor in Steller sea lions trained to perform serial dives to 50 m [52], because in all cases the animals were most probably diving within their physiological range/control. In some pinnipeds such as the elephant seal, the hepatic sinus and caval sphincter may provide a similar mechanism to limit venous return to the right atrium, subsequently restricting pulmonary cardiac output although through a different anatomical structure [54,55].

## 5. The new hypothesis: possible traits to induce a complete pulmonary functional shunt (full ventilation perfusion mismatch) in cetaceans through differential distribution of alveolar collapse and precise control of cardiac output

**Figure 2.**
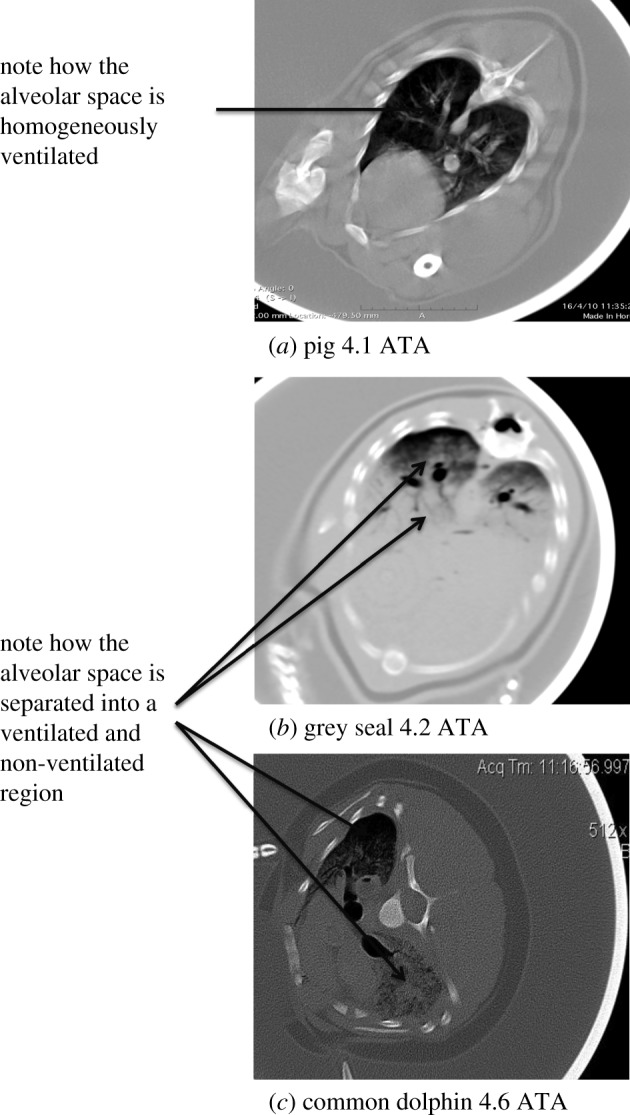
Computed tomographic image of (*a*) pig, (*b*) grey seal and (*c*) common dolphin pressurized in a hyperbaric chamber at 4–4.5 ATA. Note the difference of pulmonary gas distribution and location in terrestrial versus marine species. In the seal and dolphin, air predominates at the upper portion of the lung in the uppermost side of the body. In the pig, it remains uniformly distributed despite being under similar pressure and axial rotation. Data published here generated during the Moore *et al.* [28] study. Image slices are selected to show the anatomy at the mid-thoracic level behind tracheal bifurcation and all include the heart.

**Figure 3.**
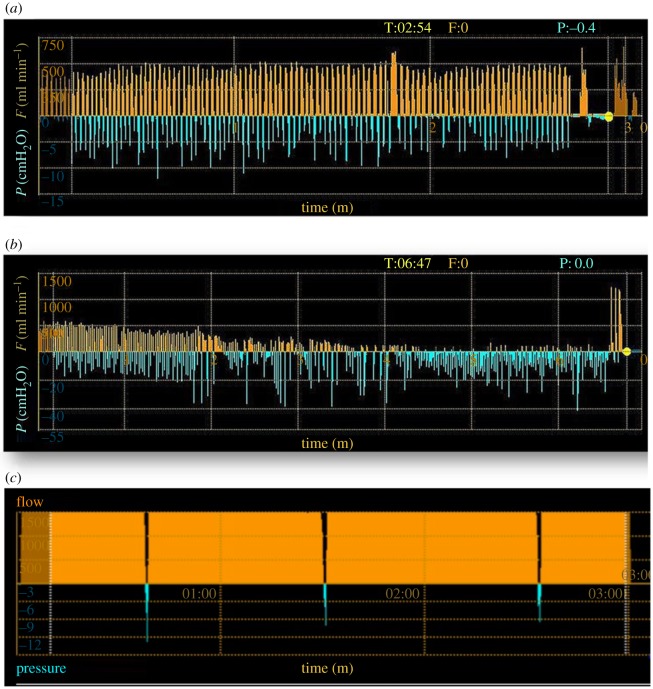
The Chartis System (PulmonX Inc.) is a medical tool to assess the level of collateral ventilation in human patients. By bronchoscopically occluding a conducting airway, and only allowing one-way flow out of the section of the lung that has been occluded, the level of collateral ventilation can be assessed. The orange pattern shows the expired flow (ml min^−1^) out of the occluded region. (*a*) In a human lung with collateral ventilation, there should be some level of flow through that section of the lung. (*b*) In a human lung without collateral ventilation, there is a decrease in the flow with each breath as the lung section collapses. In humans, flow is generally approximately 600–1000 ml min^−1^ for collateral ventilation, but in the dolphin (*c*) the collateral ventilation was greater than the machine could measure. The continuous flow out of the occluded region without any evident progressive decline indicated a high level of collateral ventilation. The blue pattern shows the negative intrapleural pressure (cmH_2_O) during each breath and indicates the quality of the occlusion by the balloon (reproduced from fig. 1*a,b* of Shah & Herth [65]).

The present knowledge of physiological and anatomical adaptations in cetaceans includes the following factors: (1) volitional control and extreme flexibility of 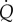 (heart rate and stroke volume) [56–59]; (2) highly compliant lung parenchyma in association with collateral ventilation allowing air to escape from alveoli to alveoli to uppermost areas, creating a differential parenchymal collapse at depth as evidenced by CT images of seal and dolphin specimens in a hyperbaric chamber (figure 2); (3) the presence of reinforced airways favouring parenchymal collapse and air accumulation in respiratory dead space [1]; (4) the absence of hypoxic pulmonary vasoconstriction response in studied marine mammals [60]; (5) the presence of myoelastic sphincters located between the exchange and non-exchange portions of the respiratory tract and/or the presence of parenchymal/pleural smooth muscular bundles/cells [2,3,16,61]; and (6) evidence of intrapulmonary arteriovenous shunts/anastomoses in some deep-diving species [3]. Based on these factors, we hypothesize that cetaceans may be able to create a full respiratory shunt even at shallow depths not fully dependent on hydrostatic compression.

Trans-alveolar–septal pulmonary venous–arterial anastomoses have been described in several cetaceans at the level of the alveoli [3,62], and a similar structure is also present in Baird's beaked whales, where an arterialvenous plexus is referenced in the interlobular stroma around the alveoli [16], although clear anatomical or histological description or evidence of real direct shunting capability have never been demonstrated. In any case, based on the previous mechanisms described, direct arterialvenous anastomosis would not be needed to create an extreme *V*_A_/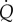 mismatch in the lung. Ventilated areas would be towards the region closest to the surface (lowest pressure), which would receive minimal perfusion due to the effect of gravity and higher vascular resistance due to distended alveoli. Blood flow would be favoured to non-ventilated areas (atelectasic) regions where the vascular resistance would be minimal due to lack of capillary stretching induced by alveolar distension and the hypoxic vasodilation [60].

Avoidance of direct pulmonary arteriovenous shunts and operating with a functional bypass could be a wise alternative for diving species that could be at risk of bubble formation in the blood. In fact intra-cardiac communications or arteriovenous shunting of lung circulation has been directly associated with increased risk of suffering DCS after diving due to elimination of the bubble-filtering capability of the human lung [63].

Extremely high collateral ventilation has been measured in excised lungs of two different cetacean species, (a white-sided dolphin and a pilot whale) [1,64], as well as *in vivo* in a bottlenose dolphin using a Chartis System (PulmonX Inc., Redwood City, CA, USA) through bronchoscopy (figure 3). The lack of lung lobulation in cetaceans would minimize air trapping and facilitate separation of a ventilated (upper region) and non-ventilated (lower region) region that would be differentially perfused [1]. Such dramatic separation of a recruited and collapsed region is apparent during hyperbaric CT studies in the compressed lung of both pinnipeds and cetaceans, but not in a terrestrial mammal model (pig) (figure 2).

**Figure 4.**
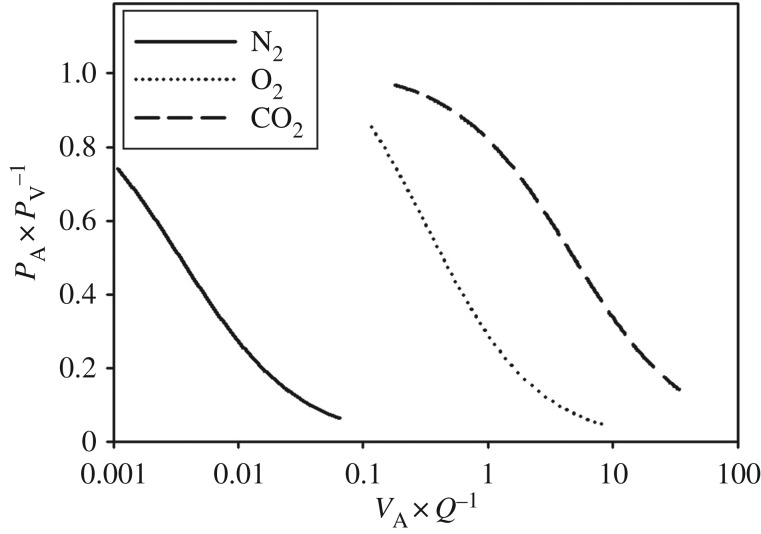
Variation in 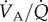 changes the relative alveolar (*P*_A_) and end-capillary pulmonary venous (*P*_V_) tension for each gas. Thus, *P*_A_ × *P*_V_^−1^ represents the level of exchange, with 1 being perfect matching and 0 being no gas exchange (i.e. full pulmonary shunt). Reproduced from Farhi [67] and modified by the authors. Note how O_2_ and CO_2_ exchange is favoured in areas of high 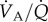 ratios, while N_2_ is exchanged in regions with lower 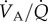. Thus, matching 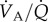 would allow O_2_ and CO_2_ exchange with minimal or no N_2_ exchange.

A functional shunt provides a possibility to selectively manage 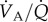 by varying 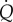. Limited perfusion of the uppermost ventilated areas of the lung could occur at depth by an increase of 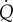. This mechanism would allow exchange of O_2_ and CO_2_ with minimal exchange of N_2_ provided the 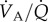 ratio is kept elevated [66]. This selective exchange of different gas species is caused by differences in gas solubility, which alters whether a gas is perfusion or diffusion limited. For example, a high 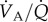 would favour O_2_ and CO_2_ exchange, while reducing, or possibly even reversing N_2_ exchange (figure 4). In the erect human, the effect of gravity has shown to result in a 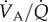 mismatch, which results in preferential gas exchange for O_2_ and CO_2_ in regions where 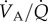 is high and for N_2_ in regions where 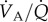 is low [68]. In cetaceans, enhanced collateral ventilation would cause pulmonary gas to remain in the upper portions of the lung (figure 2), and a hypoxia-induced vasodilatation of the lower compressed/collapsed regions and effect of gravity would create two distinct\regions of either high or low 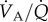 (see electronic supplementary material, S2). Consequently, cetaceans at depth may have the ability to alter the 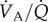 ratio to reduce inert gas uptake while still exchanging some O_2_ and CO_2_ (figure 4) [66–68]. This mechanism may help explain why cetaceans alter heart rate and lung perfusion at depth, in order to access pulmonary O_2_ during prolonged dives or increased exercise demands [69–71], with minimal uptake of N_2_. In some cases, these periods of elevated cardiac frequency have been referred to as cardiac arrhythmias [70]. In fact the elevation of heart rate is commonly observed from the beginning of the ascent phase in studied marine mammals [8,33,56,70–72]. In this context, the myoelastic pulmonary sphincters observed in cetaceans may help to retain the air in the conducting airways which limits alveolar recruitment during ascent, especially in the lower parts of the lung, thereby maintaining the 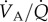 ratio to prevent N_2_ uptake during ascent. Recruitment could then occur more gradually through collateral ventilation from uppermost, highly ventilated/poorly perfused areas (alveolar dead space) progressively into lower areas with re-expansion. Increased systemic perfusion during ascent would also serve to distribute any dissolved N_2_ over a greater body volume to reduce the risk of supersaturation. Closer to the surface, the myoelastic sphincters would open, causing full alveolar recruitment through the tracheobronchial tree with a simultaneous increase of 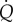 towards increasing 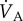 and 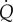 matching, maximizing gas exchange.

This mechanism would also explain the presence of GE in stranded/bycaught cetaceans. Any disruption leading to abnormally high 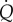 through non-collapsed regions (e.g. through prolonged elevation of sympathetic tone and a cessation of diving bradycardia and increase of 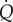) could lead to an increased N_2_ uptake at depth, resulting in bubble formation and DCS during or after ascent (depending on the amount of gas dissolved), similar to that described for sea turtles. While both 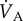 and 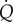 are essential for gas exchange, their relative importance for a certain gas varies with the 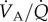 ratio. In terrestrial mammals 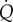 is in fact one of the most relevant factors when predicting DCS risk [37], but in marine mammals the interaction between 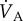 and 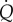 and variation in the 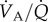 ratios may be critical. This could be an additional reason for why heart rate and 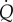 seem to be under volitional control in these species [56–58].

In the case of beaked whales exposed to sonar, a highly evasive species that appear to dive at the very limit of a breath-hold diver (and so, being even more sensitive to acoustic stress compared to other marine mammal species), a strong evasive response may result in increased activity, resulting in hypercapnia and elevated lung perfusion. Both increased 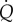 and elevated CO_2_ have been suggested to increase the risk of GE [37,73–76]. In addition, increased stress may result in failure of normal physiological mechanisms that help reduce gas exchange similar to that proposed in the pulmonary arterial system in sea turtles [50]. Variation in dive behaviour has already been considered as a possible cause for the stranding in these species [77]. In a more recent study, the physiological aspects were considered, and the study concluded that changes in dive behaviour and physiology were the most probably reasons for GE and sonar-associated mass strandings [45]. In summary, gas bubble formation, hypoxia, acidosis and arrhythmias (especially those leading to an increase of cardiac output) were considered to result from extended dive duration and time at depth, changes in ascent or descent rates, or increased duration of surface intervals. We consider that even a strong evasive response, resulting in elevated activity, could lead to significant hypercapnia and increased lung perfusion even through ventilated areas. This would reduce the 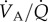 ratio at this level, resulting in increased N_2_ uptake. Increased PCO_2_ and PN_2_ may both increase the risk of GE and DCS symptoms. Indeed, bycaught cetaceans and seals were shown to have elevated PCO_2_ and PN_2_ [49].

While the evidence for the proposed mechanism is from a range of species, it has been used to propose how cetaceans manage pulmonary ventilation–perfusion to regulate pulmonary exchange of gases. A similar mechanism may be present in other diving animals, and the general mechanism would be that passive lung collapse is not required to avoid N_2_ uptake. If the lung is ventilated but not perfused, there will not be any gas exchange. Consequently, the ventilation–perfusion matching (distribution) is the critical component in our proposed mechanism, while the total volume of air in the alveolar space or the total volume of blood reaching the lung is less important. For example, diving sea turtles appear to use a specific mechanism which includes a muscular sphincter that regulates pulmonary blood flow [40], and birds may use different anatomical or functional mechanisms to manage the ventilation–perfusion to avoid DCS.

Most recent physiological work has been focused on the potential for N_2_ supersaturation, and how changes in dive behaviour could lead to bubble formation and increased risk of DCS. Alternative routes for inducing pulmonary shunt at a shallower depth should be considered for cetaceans, requiring future work to fully understand how these animals dive to such great depths and why they occasionally can suffer from GE whenever their anatomical, physiological and behavioural compensatory mechanisms are disrupted. *In vitro* studies measuring the response in live tissues, improved anatomical details, development of novel biologging capacities for obtaining measurements on free-ranging animals and medical imaging studies using trained animals under voluntary control will provide exciting avenues to investigate the cardiopulmonary adaptations hypothesized in the current perspective.

## Supplementary Material

S2-Diapositiva1

## Data accessibility

This article has no additional data.
