# Supplementary figures and images for: Pulmonary ventilation–perfusion mismatch: a novel hypothesis for how diving vertebrates may avoid the bends
Source: Proc Biol Sci. 2018 Apr 25;285(1877):20180482. doi: 10.1098/rspb.2018.0482 (PMC5936736; doi:10.1098/rspb.2018.0482)

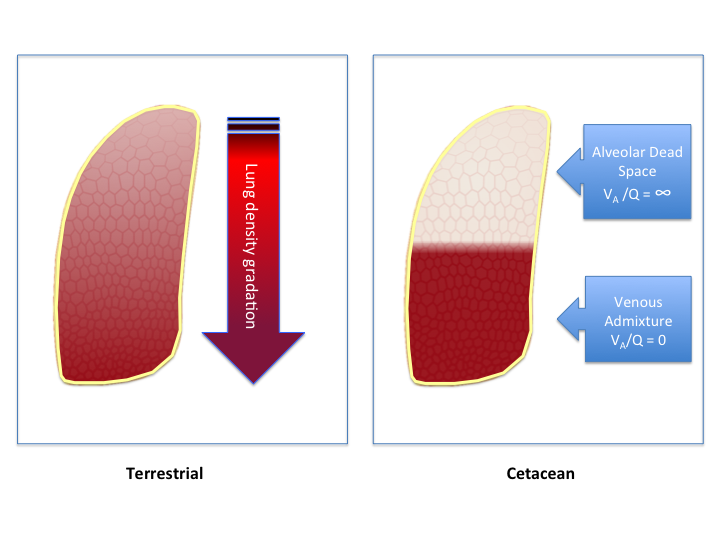

Supplement: S2-Diapositiva1 [file rspb20180482supp1.tiff]
